# Editorial: Advances in research on aging in female infertility and pathologic pregnancy

**DOI:** 10.3389/fphys.2024.1413425

**Published:** 2024-04-19

**Authors:** Bin Liu, Gendie Lash, Fengxiang Wei, Priyadarshini Pantham

**Affiliations:** ^1^ Maternal and Child Health Research Institute, Shenzhen Baoan Women’s and Children’s Hospital, Shenzhen, Guangdong, China; ^2^ Division of Uterine Vascular Biology, Guangzhou Institute of Pediatrics, Guangzhou Women and Children’s Medical Center, Guangzhou Medical University, Guangzhou, China; ^3^ Genetics Laboratory, Longgang District Maternity and Child Healthcare Hospital of Shenzhen City, Shenzhen, Guangdong, China; ^4^ Department of Obstetrics, Gynecology, and Reproductive Sciences, University of California, San Diego, La Jolla, CA, United States

**Keywords:** advanced age, female infertility, pathological pregnancy, ovarian diseases, peripheral index

Older women of childbearing age have increased risks of infertility and pregnancy complications. These impose greater challenges to woman’s reproductive health because of the global shift in population structure toward older age groups. Female reproductive health includes normal ovarian and endometrial function, successful fertilization and embryo implantation, and fetal growth. Various environmental and physiological factors contribute to declined fertility and pathological pregnancy in females. Researchers have conducted a large amount of functional and multiple-omics studies in reproductive tissues and organs in respect of various reproductive health issues including endometritis, polycystic ovarian syndrome (PCOS), recurrent miscarriage, preeclampsia, preterm birth, and various age-associated reproductive disorders. However, the mechanisms of these disorders remain largely unknown, and the methods of diagnosis and treatment of related diseases needs be significantly improved.

In this Research Topic, we aimed to collect manuscripts that would contribute to our understanding of age-associated reproductive disorders and their effects on infertility.

This Research Topic contains 12 manuscripts covering both original articles and reviews. The collected articles highlight the importance of ovarian aging in female infertility and pathological pregnancy. Zhang et al. reviewed recent developments in the mechanisms underlying primordial follicle activation and their clinical applications for improving the pregnancy rate for premature ovarian failure patients (POF). In this review, the authors focused on several signaling pathways between oocytes and granulosa cells and two waves of primordial follicle activation, which is determined by PI3K signaling in the oocytes and mTOR signaling in pre-granulosa cells, respectively. Finally, they introduced the application of *in vitro* activation (IVA) of primordial follicles for POF patients. In addition, Hu et al. discussed the latest *in vitro* techniques for fertility preservation of patients with cancer and POF, mainly on ovarian organ function reconstruction, including *in vitro* culture of oocytes, female germ cell induction from pluripotent stem cells *in vitro*, artificial ovary construction, and construction of ovarian-related organoids. Tang et al. summarized the roles of macrophages both in normal ovarian function and in the process of ovarian senescence, especially macrophage polarization, and inflammation and fibrosis within the aging ovary. In a mouse model of PCOS, Shah et al. found that quercetin can reverse the molecular, functional and morphological abnormalities brought up by letrozole, particularly the improvement of reproduction in PCOS. The authors thought that this flavonoid molecule may act as a promising medicine for human PCOS. Low response to controlled ovarian stimulation is a crucial concern in poor responders to ovarian stimulation and patients with poor-quality embryos who are undergoing assisted reproductive technology. Takeuchi et al. developed an original protocol using medroxyprogesterone acetate and high-dose gonadotropin, and found that it increased the number of collected MII oocytes, 2PN zygotes, blastocysts with high quality, and live birth rates in both poor responders to ovarian stimulation and those with poor-quality embryos.

Pathological pregnancy is a major concern for mothers of advanced age. Wang et al. reviewed the basic function of exosome lncRNA and its roles in endometrial tolerance, embryo implantation, and immune tolerance at the maternal-fetal interface. Moreover, its associations with preeclampsia, gestational diabetes mellitus, and recurrent pregnancy loss were discussed. Diagnoses of pathological pregnancy was also involved in this Research Topic. Zhang et al. used a combination of uterocervical angle (UCA) and cervical length (CL) in early and mid-pregnancy and achieved a higher probability to predict preterm birth than CL or UCA alone. Meanwhile, Ripani et al. studied the side effects of CFTR modulators on the child of a successful pregnancy in one 30-year-old Italian with cystic fibrosis (CF, F508del/R334W) and observed no significant side effects in this child.

Aging-related risk factors of female infertility and pathological pregnancy is another area address in this Research Topic. Liu et al. analyzed the long-term trend of uterine fibroid burden in Chinese women from 1990 to 2019 and found that the age-standardized rates were all on the ascending trend, with the greatest increase in the age-standardized mortality rate (53% of annual increase). They concluded that uterine fibroids are still the most common benign gynecological tumors in women, and more work on social health prevention and control should be applied. Yong et al. found that age-related CPT1B decline may explain decreased metabolic function in placenta, and therefore may be related to pregnancy complications in women with advanced age, particularly in those who are overweight. In addition to uterine fibroids and obesity, there is an increasing number of patients experiencing infertility due to chronic salpingitis after various of infections. Zhang et al. found that extracellular vesicles derived from human umbilical cord mesenchymal stem cells can relieve salpingitis by promoting M1-to-M2 transformation of macrophages in a mouse model. Urinary incontinence (UI) is linked to obesity and childbirth in aged women. Hong et al. retrospectively analyzed the NHANES data and found that total muscle-to-fat ratio showed a negative association with UI, and this association might be mediated by the peripheral index lymphocyte count.

We hope this Research Topic will be useful for researchers and clinicians alike to help further studies. More functional and population studies on aging in female infertility and pathological pregnancy are warranted.

